# Dividing the Pedicle: Subpectoral Breast Augmentation Beneath Bilateral Transverse Upper Gracilis Myocutaneous Free Flaps

**Published:** 2018-05-08

**Authors:** Kevin T. Jubbal, Dmitry Zavlin, Jessica F. Rose, Anthony Echo

**Affiliations:** ^a^Department of Plastic Surgery, Loma Linda University Medical Center, Loma Linda, Calif; ^b^Institute for Reconstructive Surgery, Houston Methodist Hospital, Weill Cornell Medicine, Houston, Tex

**Keywords:** breast, free tissue flaps, breast implantation, microsurgery, mammaplasty

## DESCRIPTION

**Figure 1:**
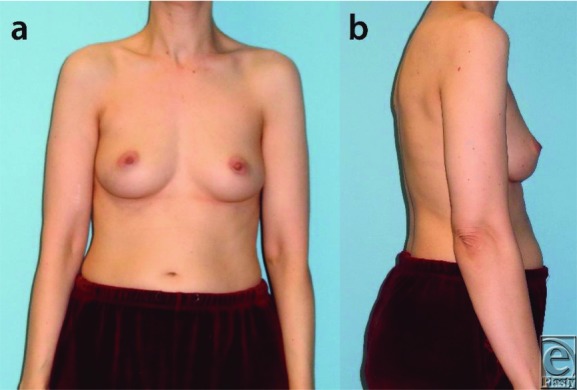
Preoperative frontal (a) and lateral (b) views of the torso. The relative size of the left breast is smaller than that of the right side, with asymmetric nipple-areola complex position.

**Figure 2:**
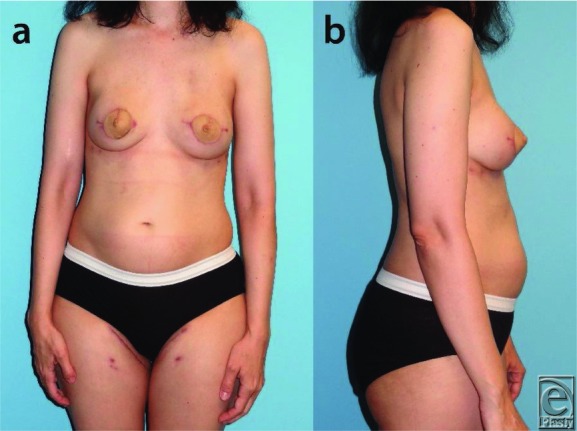
Three-month postoperative frontal (a) and lateral (b) views after bilateral mastectomies and immediate reconstruction with transverse upper gracilis flaps.

**Figure 3:**
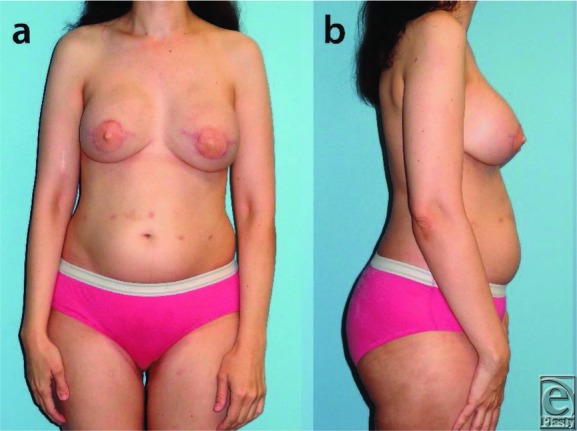
Thirteen-month postoperative frontal (a) and lateral (b) views after breast revision including bilateral breast augmentation with implants and division of transverse upper gracilis flap pedicles bilaterally.

A 45-year-old female patient with left-sided lobular carcinoma in situ (Fig 1) underwent bilateral mastectomies with immediate free transverse upper gracilis (TUG) flap breast reconstructions (Fig 2). Desiring augmentation and improvements in symmetry 6 months postoperatively, she received 205-mL implants in each subpectoral plane after bilateral pedicle division. No complications occurred, and the flaps remained soft and viable without fat necrosis (Fig 3).

## QUESTIONS

1. What are the challenges of breast augmentation after autologous reconstruction?
2. What factors determine survival of free flaps?
3. What has often been suggested in literature to increase flap safety during augmentation with pedicle division?
4. Are there recommended wait periods after free tissue breast reconstruction before pedicle divisions can be safely performed?

## DISCUSSION

Breast reconstruction combining implants with autologous tissue has been performed in the past. Combining autologous and implant-based methods for breast reconstruction allows for reduction in the risk of wound breakdown and infection^1^ in addition to improved aesthetic outcomes. Some patients, such as the one presented in this case, may also desire larger breasts, which are subject to constraints of autologous tissues based on patient-specific variables. However, secondary augmentation after autologous reconstruction has been subject to limitations of technique. These additional challenges include access incision, choice of plane, and planning around the vascular pedicle. Division of the pedicle in this case was necessary for optimal placement of the implant, which provided adequate volume of the breasts.

Survival of free flaps is initially based on the vascular pedicle, but other factors contribute to survival after a period of time, including closing of arteriovenous shunts,^2^ conditioning tissues for hypoxia,^3^ improving blood flow to the flap via vasodilation of existing vessels,^4^ and angiogenesis providing new vascular supply. It is through these methods that the pedicle can be divided in time without flap compromise.

Although multiple clinical studies and reports demonstrate reliance on the vascular pedicle, there have been reports of flap survival after early division or compromise of the vascular pedicle.^5^ Other methods of breast reconstruction such as the transverse rectus abdominis myocutaneous (TRAM) flap delay have demonstrated the utility of flap delay to increase flap reliability,^6^ but flap delay with breast reconstruction is generally performed over the course of weeks prior to final placement. Studies on the deep inferior epigastric artery perforator flap, a fasciocutaneous flap commonly used in breast reconstruction, suggest that pedicle dependency is maintained in the long term.^7^

However, Enajat et al^5^ demonstrated survival of a superficial inferior epigastric artery flap after inadvertently avulsing the vascular pedicle on the 11th postoperative day. These differences may be due to varying properties of revascularization in myocutaneous versus fasciocutaneous flaps. Myocutaneous flaps have demonstrated long-term dependence on their pedicle for vascular supply.^8^ Nevertheless, we demonstrate survival of a freely transferred myocutaneous flap after 6 months from initial inset. Controversy still exists regarding the dependency of the vascular supply on the pedicle and when neovascularization from the periphery adequately compensates. Further studies will be necessary to determine the minimum time necessary to prevent flap loss or fat necrosis following pedicle division.

In this case, the patient desired additional volume that autologous tissue was not able to provide. Therefore, alloplastic implantation was pursued and division of the pedicle provided the advantage of allowing for proper subpectoral placement and medialization of the implant. We demonstrate that an alloplastic implant can be added at a second stage with autologous breast reconstruction utilizing TUG free flap, and the vascular pedicle can be safely divided after 6 months to allow for optimal placement of the implant.
